# Municipal Crèches

**Published:** 1904-08-13

**Authors:** 


### Municipal Crèches.

The long-standing dispute between those who advocate and those who oppose an extension of the province of State or municipal interference may, in the world of theory, be continued with unabated strenuousness, but in certain departments of practical life circumstances have settled the question by rendering such interference imperative. More particularly is this true in regard to what was formerly considered to be purely a matter of family or personal concern—namely, the education and protection of children. Even amongst those who oppose this modern tendency there are few who urge an actual retreat, though their voices may be raised against a further advance. It is recognised generally that the State has a large interest in the mental and physical development of its future citizens, and that it is justified in taking steps to protect this interest. There is one group of children for whom it is obvious some special measure of protection is needed, namely, the children whose mothers daily leave their homes to work for many hours in a workshop or factory. It is for these the crèche is suggested. The child would be received in the morning, fed and nursed during the day, and handed back to its mother in the evening. The alternative to this proceeding is to leave the child in the care of a neighbour or friend or to employ a diminutive nurse girl, with all the risks that this involves.

It is beyond dispute that many children so situated are neglected and are even badly treated. And it may be taken as certain that as the facts become known the demand for the provision of some form of public nurseries will become irresistible. Whether these should be organised and controlled by the municipal authorities or should be left to private enterprise or the promptings of charity may be open to argument, but that the necessity for such institutions will be recognised cannot be doubted. The number of married women employed in factories is a very large one, and is increasing rather than diminishing. It may be that a wise policy would, as urged in the House of Commons a few days ago, prevent such employment, but public opinion has certainly not reached this stage—at least in the meantime. There remains, however, the fact that the children of such mothers must almost of necessity be reared under conditions which imperil their value to the State. Hence if those who argue that this position may be met by individual effort can translate their contention into practice now is the time for action. For there are obvious signs that ere long the problem will be beyond their control. Thus, at a recent conference summoned by the London County Council to consider the administration of the Public Health Act of 1891, the delegates, appointed by some 25 sanitary authorities, unanimously agreed, *inter alia*, to a resolution declaring it desirable that the Borough Councils should be empowered to establish crèches for the reception of young children and to make reasonable charges for the accommodation and food so provided. It was also decided to request the County Council to obtain from Parliament the authority necessary to confer such discretionary power upon the Borough Councils. There will doubtless be not a few ready to denounce such a proposal, and probably their opposition will be sharpened by the announcement that Mr. Seddon intends to propose a similar scheme to the New Zealand Parliament. In their support they may urge the usual arguments of the non-interference school and may protest that already some 60,000 children are wholly dependent on the rates. All this will in all probability be entirely in vain. If crèches are not supplied by private enterprise, and of this we see no prospect, they must be organised by the local authorities. Whether they can be made self-supporting or not is a matter for experience to decide; but in any case they are a necessity of present-day social conditions. It is only necessary to read the address delivered by Sir George Kekewich to the Royal Institute of Public Health to appreciate the serious prejudice to educational, industrial, and economic interests arising from the unsatisfactory conditions in which many children are reared in their early years. The municipal crèche will meet this defect in a limited, but not unimportant field. Hence the scheme may be commended as one deserving of sympathy. If carried into action it will help to meet an acknowledged evil, and even for those who would render this evil impossible by more radical measures there is merit in the proposal to the extent that it does something to protect infants until the time when public opinion will recognise that national welfare demands the presence of the mother in the home and not in the workshop.

